# Prevalence and subtype identification of *Blastocystis* isolated from humans in Ahvaz, Southwestern Iran 

**Published:** 2017

**Authors:** Roya Salehi, Ali Haghighi, C. Rune Stensvold, Farnaz Kheirandish, Eznelloah Azargashb, Saber Raeghi, Cobra Kohansal, Fares Bahrami

**Affiliations:** 1 *International Branch, Shahid Beheshti University of Medical Sciences, Tehran, Iran*; 2 *Department of Medical Parasitology and Mycology, School of Medicine, Shahid Beheshti University of Medical Sciences, Tehran, Iran*; 3 *Department of Microbiology and Infection Control, Statens Serum Institute, Artillerivej 5, DK-2300 Copenhagen S, Denmark*; 4 *Department of Medical Parasitology and Mycology, School of Medicine, Lorestan University of Medical Sciences, Khorramabad, Iran*; 5 *Department of Community Medicine, School of Medicine, Shahid Beheshti University of Medical Sciences, Tehran, Iran*; 6 *Department of Laboratory Science, Faculty of Paramedical Sciences, Maragheh, Iran. *; 7 *Department of Medical Parasitology, School of Medicine, Jondishapour University of Medical Sciences, Ahvaz,Iran*

**Keywords:** Blastocystis, Prevalence, Subtypes. South western Iran

## Abstract

**Aim:**

The aim of the present study was to determine the prevalence and subtype distribution of Blastocystis and its relation with demographic data and symptoms in humans referred to medical centers in Ahvaz 2014-2015.

**Background:**

Infections with intestinal parasites are one of the most important threats to human health worldwide, especially in tropical and subtropical areas. Blastocystis sp. is a common parasite of humans with a vast variety of non-human hosts. We aimed to study the prevalence and subtypes of Blastocystis sp. in individuals referred to medical laboratories in Ahvaz city, southwest Iran.

**Methods:**

From September 2014 to September 2015, 618 stool samples were collected from 16 medical laboratories in Ahvaz, and examined using direct wet mount, formalin-ether concentration, a modified version of the Ziehl–Neelsen staining technique, and cultivation in xenic HSr + S medium. Subtypes of positive Blastocysts sp. were obtained using the “barcoding” method. The results were analyzed using SPSS software, version 16, with Chi-square and Fisher’s exact test.

**Results:**

Totally, 325 (52.6%) of the referred individuals were men and 293 (47.4%) were women. Blastocystis sp. was observed in 146 (23.6%) samples. Co-infections with other intestinal parasites were found in 32 (5.17%) cases. Out of the 146 positive isolates, 20.83%, 20.83% and 58.34% belonged to ST1, ST2, ST3 respectively.

**Conclusion:**

Blastocystis sp. was quite common in the study population, with a carrier rate corresponding to nearly one in every four individuals. The subtype distribution identified in the present study was largely identical to that reported from other studies in Iran, with ST3 being the most common.

## Introduction

*Blastocystis* is a common anaerobic unicellular eukaryotic parasite of humans with a large variety of non-human hosts with a more or less global distribution. The genus comprises at least 17 ribosomal lineages, the so-called “subtypes”, which are arguably separate species (1). Nine of these subtypes, ST1-ST9, have been detected in humans, with ST1-ST4 being the most common (2). Molecular epidemiological surveys have been carried out in several countries to elucidate the genetic diversity of *Blastocystis* in different hosts, primarily to identify the level of host specificity, the possibility of zoonotic transmission, and whether certain subtypes could be linked to diseases in humans (1). However, only few countries outside Europe have published data on the genetic diversity of *Blastocystis* in different hosts (3, 4); Iran is among these countries. 

To our knowledge, few Iranian studies have been published to date aiming to elucidate the distribution of *Blastocystis* subtypes in humans (5-14). These studies used different methodologies for identifying and differentiating *Blastocystis* subtypes. State-of-the-art subtyping of *Blastocystis* involves barcoding its original methods to detect *Blastocystis* subtypes (15). We aimed to expand the knowledge on *Blastocystis* subtypes existing in humans in southwest of Iran using state-of-the-art subtyping. 

## Methods

**Subjects**

Prior to the sample collection, all participants were informed about the procedures. After taking a written consent, a personal information questionnaire was administered to each participant to inquire about age, sex, signs and symptoms, such as abdominal pain, diarrhea, dysentery, vomiting, nausea and constipation. The names of the admitted individuals in the medical laboratories and the results of used methods (Direct slide smear, culture and PCR) were written daily in check-list.

A total of 618 stool samples were collected from individuals referred to 16 medical laboratories of Ahvaz over a period of one year from September 2014 to September 2015. 

**Parasitological and Statistical Analysis**

 All 618 fecal samples were examined by direct smear (wet mount with Lugol’s staining), formalin ether concentration technique, Ziehl-Neelsen and trichrome staining in order to enable detection of Cryptosporidium spp. and *Entamoeba*. sp, respectively, and were also processed by xenic in vitro culture in HSr +S medium [Horse serum, ringer & starch rice (Razi Serum Institute, Iran)] (16). After 5-7 days, sediments of cultures were studied by microscopic examination. Data were analyzed using SPSS software, version 16 (SPSS, Chicago, IL, USA), with Chi-square and Fisher’s exact test.

**DNA extraction and PCR amplification**

After 5-7 days of cultivation, DNA of positive cultures were extracted from 200 µLit of the HSr + S culture medium using a commercial DNA extraction kit (Yekta-Tajhiz Azma stool mini kit, Iran) according to the manufacturer’s instructions. DNA was also extracted from stool deemed positive for Blastocystis by microscopy. A 620 bp fragment from 18S rRNA gene was amplified using the DNA barcoding method using RD5 and BhRDr primers as previously described (15). PCR was performed using the Taq DNA Polymerase Master Mix Red (Amplicon, Denmark). The reaction mixture contained 5 µL of distilled water, 7.5 µL master mix, 20 pmoL forward and reverse primers and about 100-500 ng/µL of extracted DNA in a final volume of 15 µL. DNA from a known Blastocystis and a blank containing all PCR reagents but no DNA were included in each set of PCR as positive and negative controls, respectively. PCR products were electrophoresed and visualized with 1.5% agarose gels stained with ethidium bromide.

**Sequence analysis and accessions**

Ab1 files available from sequencing were manually edited and sequences were queried using the standard nucleotide BLAST algorithm provided by NCBI (http://www.ncbi.nlm.nih.gov/), the Blastocystis subtype [18S rRNA] and Sequence Typing (MLST) database (http://pubmlst.org/blastocystis/), to obtain information on subtype and subtype alleles, whenever applicable (17). The nucleotide sequence of 24 reported data in the present study were submitted to the GenBank/EMBL/DDBJ database under accession number KY312690 to KY312705 and MF072942 to MF072949.

**Ethical clearance**

All procedures of this study were approved by the Ethics Committee of the Shahid Beheshti University of Medical Science (SBMU), Iran, before the beginning of the study. All participants were informed about the study procedures and written informed consents were obtained from all of them prior to sample collection. 

**Table 1:** Frequency of *Blastocystis *in 16 medical laboratories in Ahvaz, southwest Iran

Medical laboratory	No. of Samples	Positive N (Percent)	Negative N (Percent)
Baghaii hospital	25	13 (52.0)	12 (48.0)
Abozar	43	9 (21.0)	34 (79.0)
Pastour	38	6 (15.7)	32 (84.3)
Golestan	42	8 (19.0)	34 (81.0)
Razi	44	9 (20.4)	35 (79.6)
Imam Khomeini	24	5 (21.0)	19 (79.0)
Amir al moemenin	31	11 (35.4)	20 (64.6)
Naft	54	16 (29.6)	38 (70.4)
Shahid Rajaii.	100	22 (22.0)	78 (78.0)
Jihad daneshgahi	57	7 (12.2)	50 (87.8)
DR Jalali	46	11 (24.0)	35 (76.0)
Amir Kabir	25	4 (16.0)	21 (84.0)
Mehr.	16	5 (31.2)	11 (68.8)
DR Naghash	45	7 (15.5)	38 (84.5)
Shahid Karami	23	12 (52.2)	11 (47.8)
Shafa	5	1 (20.0)	4 (80.0)
Total	618	146 (23.62)	472 (76.38)

**Table 2:** Frequency of *Blastocystis* sp. isolated from humans based on demographic variables of age, sex and season in subjects referred to the medical laboratories of Ahvaz, southwest Iran

Variables	Examined individuals (N)	Infected with *Blastocystis *N (%)	*P* value
Sex Male Female	325 293	85 (26.15%) 61 (20.81%)	0.141
Age group 10≥ 11-25 26-40 41-55 56-70 ≤71	131 78 39 87 145 138	23 (17.55) 12 (15.4) 16 (41) 22 (25.3) 38 (26.2) 35 (25.3)	0.023
Season Spring Summer Autumn Winter	152 150 164 152	47 (30.92%) 44 (29.33%) 37 (22.56%) 18 (11.84%)	0.001

## Results

Out of 618 collected stool samples, 325 (52.6%) were from men and 293 (47.4%) were from women. Samples were randomly collected from individuals referred to the 16 laboratories of Medical centers in 8 regions of Ahvaz and Blastocystis sp was seen in 146 (23.62%) samples (Table 1). Table 2 shows the frequency of positive Blastocystis isolates based on demographic variable of sex, age, and different seasons. In this study, 40.29% of the participants (249/618) were infected by one or more pathogenic or non-pathogenic intestinal parasites. Single parasites were seen in 198 (32.03%) of the specimens, while only 3 (0.48%) of the patients were infected with helminthes. Table 3 shows the prevalence of different intestinal parasites in the collected samples. Co-infections with two or three parasites were found in 32 (5.17%) of positive samples. Frequency of infection was higher in spring and summer and the correlation between season and presence of Blastocystis was significant (P≤0.001). However, no significant correlation was found between sex and infection (Table 2).

**Figure 1:**
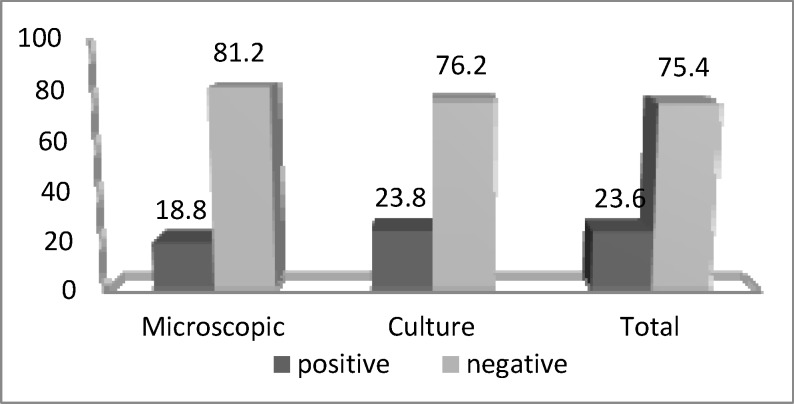
Frequency of *Blastocystis *sp. identified by microscopy and culture media from individuals who referred to the medical laboratories in Ahvaz (2014-2015

In microscopic study, *Blastosistis* sp. was seen in 116 (18.77%) samples, while 146 (23.6%) samples grew in culture media (Figure1).

Among the participants, 256 (41.42%) who were referred to the medical laboratories for checkup had no symptoms and 362 (58.58%) individuals suffered from at least one gastrointestinal symptom. In the symptomatic patients, totally 96 (26.51%) Blastocystis sp were isolated (Table 4). A significant correlation was found between stomach pain, diarrhea and Blastocystis infection (P≤0.01). 

In molecular study, all 146 (23.62%) positive culture isolates were given expected amplicon. From those positive isolates, 24 positive PCR samples were randomly sequenced. Three subtypes, including ST1 (5/20.83%), ST2 (5/20.83%), and ST3 (14/58.34%), were identified. While most patients suffered from abdominal pain and diarrhea, no significant correlation was found between symptoms and subtypes (Table 5).

**Table 3.** Frequency of intestinal parasites from individuals referred to the medicallaboratories, in Ahvaz, Khuzestan province, Southwest Iran (2014-2015)

Parasite	NO.	%
*Blastocystis*sp.	146	23.78
*Endolimax nana*	34	5.5
*Entamoeba coli*	32	5.17
*Giardia lambelia*	26	4.2
*Chilomastix mesnelii*	3	0.48
*Cryptosporidium.spp.*	2	0.32
*E.histolytica/E.dispar*	2	0.32
*Dientamoeba fragilis*	1	0.16
*Total protozoa*	246	39.80
*Hymenolepis nana*	2	0.32
*Oxyur*	1	0.16
Total parasites	249*	40.29*

* Co-infections with two or three parasites were found in 32 (5.17%) of the positive samples

**Table 4:** Frequency of *Blastocystis* sp. according to clinical manifestation among individuals who referred to the medical laboratories in Ahvaz (N=618

Clinical features	Examined individuals (N)	Infected with *Blastocystis* sp N (%)	*P.* value
Stomach pain			
Yes No	181 437	51 (28.2%) 94 (21.5%)	0.094
Diarrhea			
Yes No	61 557	22 (36.1%) 123 (22.1%)	0.014
Dysentery			
Yes No	6 612	3 (50%) 142 (23.2%)	0.145
Vomiting			
Yes No	31 586	6 (19.4%) 138 (23.5%)	0.749
Nausea			
Yes No	90 528	28 (31.1%) 117 (22.2%)	0.086
Constipation			
Yes No	18 600	5 (27.8%) 140 (23.3%)	0.876
In appetence			
Yes No	209 409	61 (29.2%) 84 (20.5%)	0.21
Group study			
Patients Asymptomatic individuals Total	362 256 618	96 (26.51%) 50 (19.53%) 146 (23.6%)	0.017

**Table 5:** Frequency of *Blastocystis* sp. according to gastrointestinal disorders and subtypes among individuals who referred to the medical laboratories in Ahvaz

P-Value	Subtypes ST1 ST2 * ST3*			Symptom*
0.486	4	3	3	Stomach pain, Inappetence
0.967	1	4	0	Stomach pain, Nausea
0.87	2	1	0	Stomach pain, Constipation
0.758	4	1	1	Stomach pain, Diarrhea
0.967	1	0	0	Stomach pain, Vomiting
0.967	0	0	0	Diarrhea, Vomiting
0.967	0	0	1	Nausea, Vomiting
0.967	3	0	0	Inappetence, Constipation
1.0	0	0	0	Dysentery
	14	5	5	Total subtypes

* Subtypes were seen sometimes in two or more symptoms

## Discussion

*Blastocystis* is the most common parasite that infects the gastrointestinal tract of humans and a wide range of animals, including mammals, birds, reptiles, and arthropods, with a worldwide distribution. The purpose of this study was to improve our understanding of the molecular epidemiology of human Blastocystis, focusing on 618 randomly stool collected from 16 medical laboratory of Ahvaz in one year. 

In the present study, the prevalence rate of *Blastocystis* was 23.62%. In developing countries, *Blastocystis* has a higher prevalence (30-50%) compared to developed countries (1.5-10%) (17). A noticeable result was obtained (30/20.54% out of 146 positive) in comparison to positive microscopy results when all studied isolates were cultivated in HSr + S medium (16). Therefore, cultivation not only increases positive samples, but the positive culture media is very useful for DNA extraction. Our findings corroborate results from other studies although it is generally assumed that sex is not a risk factor for infection with Blastocystis (18). 

We sought to elucidate the distribution of *Blastocystis* subtypes in humans in southwest of Iran. ST1, ST2 and ST3 were identified, confirming the trend observed in other studies carried out in countries outside Europe. Hence, no cases of other subtypes were found. ST4 is common in humans in Europe, but appears to be rare in countries outside Europe (19). In this study, ST3 was the most prevalent (58.34%), as the pre-dominant subtype in most parts of the world such as Japan, Pakistan, Bangladesh, Germany, Singapore, Greece, Turkey, Makkah, Thailand and Iran (2, 5, 7-9, 10, 11, 17, 19-24). It is believed that ST3 is the main human subtype and has no relation to geographic area (25-27). Moosavi and colleagues (2012) also identified ST1, ST2, and ST3 in humans; however, these authors also found a few cases of ST7 (5), which has been found sporadically in humans in other studies (4, 26, 27). ST5 is the subtype seen in cattle and pigs (28), and human infection with this subtype has been rarely reported (25, 28). Since *Blastocystis* is a zoonotic parasite, the impact of geographical terms on infection should be considered. 

There has been debate on the pathogenicity of *Blastocystis*. A few studies found that expatriates with traveler’s diarrhea had a high prevalence of *Blastocystis*, whereas some studies found that 25%–75% of those with *Blastocystis* have a history of recent foreign travel (29-32). Some studies suggest an association between the parasite and symptoms (32-34), while others do not (35, 36).

*Blastocystis* can be isolated from individuals with gastrointestinal and extra-intestinal symptoms (e.g. diarrhea, nausea, abdominal pain, bloating, vomiting or anorexia) and asymptomatic individuals with an almost equal prevalence (32). In some studies, higher prevalence can be found in asymptomatic compared to symptomatic individuals. Many researchers classify *Blastocystis* as a commensal or opportunistic pathogen (37). In this study, we compared clinical signs and infection with *Blastocystis*. A significant correlation was found between Blastocystis infection with diarrhea and stomach pain. However, no significant correlation was observed between different subtype and clinical signs. Scanlan suggested that studies about the clinical relevance of different *Blastocystis* subtypes, their virulence, and the zoonotic potential within and between humans and animals can fill the gaps of incomplete knowledge about the pathogenicity of Blastocystis (38).

Clinical symptoms are diverse, ranging from acute diarrhea to mild chronic abdominal pain. Although the parasite is noninvasive, it might complicate the pathogenicity of other invasive pathogens. The diversity in pathogenesis between variant parasite subtypes is suspected to be responsible for diverse clinical symptoms and presentations of Blastocystis infections (39).

The results of the present study implicated that more than one third of referred individuals (40.29%) were infected with one or more intestinal parasites. Our findings showed that protozoa infections (39.80%) were remarkably more common compared to helminthes infections (0.49%) and except *Blastocystis*, *Endolimax* *nana*, *Entamoeba* *coli* and *Giardia* *lamblia* were the most frequently detected protozoan parasites.

One of the limitations of our study was that PCR was not performed on DNAs extracted from negative samples by the two screening methods, both of which have reduced sensitivity compared with PCR (37, 40). To this end, it should be emphasized that the numbers of positive samples identified in the current study should by no means be interpreted as prevalence figures. We acknowledge the limitations related to methods used for *Blastocystis* screening (microscopy and culture), one of which is related to the possibility that for instance avian *Blastocystis* sp isolates may not establish in cultures kept at 37^o^C.
